# The Impact of COVID-19 Pandemic on Surgical Treatment of Resectable Non-Small Cell Lung Cancer in Greece

**DOI:** 10.3390/life13010218

**Published:** 2023-01-12

**Authors:** Ioannis Tomos, Emmanouil I. Kapetanakis, Konstantina Dimakopoulou, Thomas Raptakis, Katerina Kampoli, Anna Karakatsani, Anna Koumarianou, Spyros Papiris, Periklis Tomos

**Affiliations:** 1 2nd Pulmonary Medicine Department, School of Medicine, National and Kapodistrian University of Athens, “ATTIKON” University Hospital, 12462 Athens, Greece; 2 Department of Thoracic Surgery, School of Medicine, National and Kapodistrian University of Athens, “ATTIKON” University Hospital, 12462 Athens, Greece; 3 Department of Hygiene, School of Medicine, Epidemiology and Medical Statistics, National and Kapodistrian University of Athens, 11528 Athens, Greece; 4 Hematology-Oncology Unit, 4th Department of Internal Medicine, National and Kapodistrian University of Athens, “ATTIKON” University Hospital, 12462 Athens, Greece

**Keywords:** COVID-19, pandemic, early stage, non-small cell lung cancer, surgery, medical care

## Abstract

Background: The coronavirus disease (COVID-19) pandemic has posed an unprecedented challenge to health systems, and has significantly affected the healthcare of lung cancer patients. The aim of our study was to assess the impact of COVID-19 on early lung cancer patients’ surgical treatment. Methods: All consecutive patients with early-stage non-small cell lung cancer eligible for surgical treatment stage I/II and resectable stage III, referred to our department during the first wave of COVID-19 between February to May 2020, were included and compared with those on the exact corresponding quarter in 2019, one year before the pandemic. Waiting time to surgical treatment, increase of tumor’s size and increase on lung cancer stage were recorded and compared. All subjects were followed up for 12 months. Multiple linear and logistic regression models were applied to assess the differences in the management of the studied groups adjusting for potential confounders. Results: Sixty-one patients with early-stage lung cancer were included in the study; 28 (median age 67 years, SD: 7.1) during the pandemic and 33 (median age 67.1 years, SD: 7.5) one year earlier. A significantly longer period of waiting for treatment and an increase in tumor size were observed during the pandemic compared to before the pandemic [median time 47 days, interquartile rate (IQR): 23–100] vs. [median time 18 days, IQR: 11–23], *p* < 0.001. No significant differences were detected in the increase of the stage of lung cancer between the subgroups. Conclusion: The COVID-19 pandemic had a significant impact on surgical and oncological care, leading to significant delays on treatment and an increase in tumor size in early-stage lung cancer patients.

## 1. Introduction

The coronavirus disease 2019 (COVID-19) pandemic caused by the severe acute respiratory syndrome coronavirus 2 (SARS-CoV-2), has overwhelmed health systems worldwide, and has significantly affected the management of lung cancer patients [1,2]. So far, COVID-19 has accounted for approximately 10% of overall lung cancer deaths [3]. Indeed, patients with lung cancer have been disproportionately affected due to their increased susceptibility to SARS-CoV-2 infection, and the impact that the pandemic has had on the delivery of cancer management and the subsequent re-allocation of resources in health systems [4]. In Greece, particularly, from the beginning of the pandemic in February 2020, lockdown measures were applied while the lack of resources and mainly intensive care beds forced officials to restrict non-priority elective surgeries to create dedicated COVID-19 wards to care for infected patients and preserve hospital resources [1,5,6].

So far, it has been shown that patients with cancer have an increased risk of infection with SARS-CoV-2 [7,8,9]. Malignancy seems to represent an independent risk factor for severe COVID-19 disease [7,9,10,11]. More particularly, patients with lung cancer have an increased risk of death compared to other cancers, potentially due to their underlying pulmonary compromise and smoking history [12]. Various factors may affect the course of COVID-19 in patients with lung cancer, including immunosuppression as a consequence of cancer or of systemic anti-cancer treatment, (chemotherapy, steroids), as well as advanced age and associated cardio-respiratory comorbidities [12]. In addition, frequent hospital visits may put patients with cancer at further risk of SARS-CoV-2 [12]. Consequently, early on during the pandemic, lung cancer professionals, especially surgeons, faced the dilemma on how to best provide oncologic surgical management to their patients safely, minimizing not only the risk of SARS-CoV-2 infection but also other potential complications while taking into consideration the reduced available resources [1,12]. Lung cancer multidisciplinary teams had to decide whether to offer, modify, postpone, or even cancel treatments for such patients [12]. This naturally produced a significant psychological burden when considering the need to avoid “losing the window to diagnose and treat” and consequently, facing a detrimental effect in survival outcomes due to suboptimal treatment [1].

Unfortunately, data regarding the effects of COVID-19 and of our decisions on early-stage non-small cell lung cancer (NSCLC) patients eligible for surgical treatment are lacking. Prompt detection and treatment at an earlier stage represent the optimal approach in lung cancer patients, while surgical removal of the tumor remains one of the cornerstones in therapeutic management [13,14]. Potential delays on the delivery of surgical treatment may have catastrophic consequences for the survival of patients with early-stage and resectable NSCLC [15]. The aim of the present study was therefore to investigate the impact on the delivery of surgical treatment in patients with early stage (stage I/II) and potentially resectable stage III NSCLC in Greece.

## 2. Materials and Methods

### 2.1. Study Population

In order to assess the consequences of the pandemic on lung cancer treatment, all consecutive patients with early stage (stage I/II) and resectable stage III NSCLC, who were eligible for surgical treatment and were referred to our tertiary level university hospital during the first pandemic wave in Greece from February to May 2020 (study group) were collected and compared with patients on the exact corresponding quarter the previous year of 2019 (control group). The diagnosis and staging of lung cancer was made based on the 8th edition of the TNM Classification of Malignant Tumors and after multidisciplinary oncological council assessment of each case. Disease reevaluation on follow-up imaging was carried out by applying RECIST Criteria [16]. All patients were invited to participate in the present study at the time of their initial assessment for treatment. The study protocol was approved by the Bioethics Committee and the Institutional Review Board of Attikon University Hospital. All patients participating in this study provided a written informed consent form. All investigations conducted in this study complied with the principles expressed in the Declaration of Helsinki.

### 2.2. Clinical Data

Demographic and clinical information regarding the histologic type of lung cancer, the initial staging at first diagnosis as assessed by presenting imaging which included chest and brain computed tomography (CT) and positron-emission tomography (PET) CT scanning and the waiting time until surgical treatment were recorded in detail. All patients were submitted to a new chest/brain CT and PET CT scan just prior to the initiation of treatment. The size of the primary tumor, both in millimeters and as expressed by T in the 8th TNM system, the size and location of invaded lymph nodes and the subsequent development of new primaries or metastasis were also recorded for each patient and compared between the initial and follow up imaging procedures.

### 2.3. Statistical Analysis

Data were described using frequencies and percentages for categorical variables. Mean values and standard deviation (SD) or median and interquartile range (IQR) were used for continuous variables. The Mann–Whitney U-test and Fisher’s exact test were used to assess differences in the clinical characteristics of lung cancer patients, before and during the COVID-19 pandemic. Two-tailed *p*-values were reported. A *p*-value less than 0.05 was considered as statistically significant. Multiple linear and logistic regression models were applied to investigate the differences in time waiting for treatment (days) and the increase in the size of the tumor (yes/no) and in the stage (yes/no) between the two groups of lung cancer patients (during COVID-19 pandemic vs. before the COVID-19 pandemic), also adjusting for the potential confounding effect of age (years), sex (male/female) and lung cancer type (squamous, adenocarcinoma, other). All statistical analysis was performed using the Stata/SE 10.0 for Windows statistical package (Stata Corp LP Lakeway Drive, College Station, TX, USA).

## 3. Results

### 3.1. Demographic and Clinical Characteristics of the Study Patients

**Figure 1:**
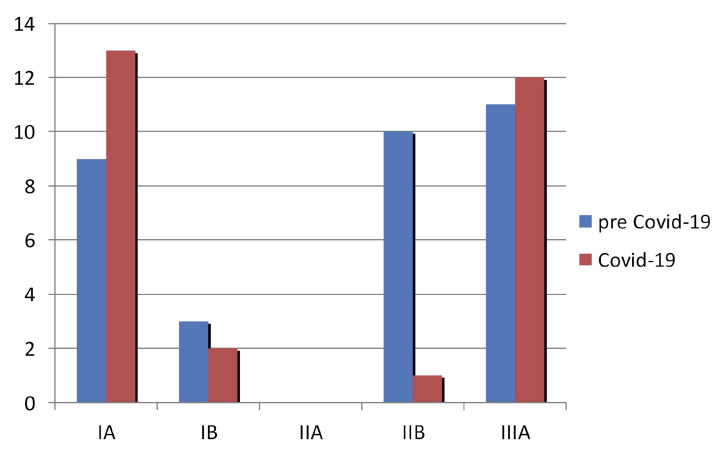
The distribution of stages of NSCLC between the two patients’ groups.

In total, 61 patients with early-stage lung cancer were included in the study; 28 (mean age 67 years, SD: 7.1) managed during the first wave of the pandemic in 2020 and 33 (mean age 67.1 years, SD: 7.5) managed one year earlier at the exact corresponding quarter in 2019. The demographic and clinical characteristics of the patients are shown in Table 1. Eighty-two percent of our cohort patients were male with the distribution amongst the study groups being comparable, with 84.8% of patients in the control group being male versus 78.6% in the study group. Patients underwent surgery, either a lobectomy or anatomical segmentectomy. In the cohort, adenocarcinoma was the most frequent type of lung cancer followed by squamous cell, while rarely, large cell lung cancer and carcinoid were detected (Table 1). The distribution of stages of NSCLC between the two groups of patients is also shown in Figure 1. As is shown, this study included a variety of stages from IA to resectable IIIA. During the pandemic, one patient died while waiting for treatment. All patients were followed up for one year at pre-determined intervals (one month, six months and one year) by attending either a physical or by a virtual/telemedicine outpatient clinic.

### 3.2. Consequences regarding the Waiting Time to Surgical Treatment

The COVID-19 pandemic had a major impact regarding surgical treatment, as it affected the waiting time of lung cancer patients to surgery. More precisely, a significantly longer period of waiting for treatment was observed during the pandemic compared to one year earlier [median time 47 days, interquartile rate (IQR): 23–100] vs. [median time 18 days, IQR: 11–23], *p* < 0.001 (Table 2).

### 3.3. Consequences regarding Disease Progression

A significant increase in the size of the tumor as expressed by either T or N of the TNM classification was observed between the two groups, *p* = 0.012. During the pandemic, eight patients developed an increase in the size of their tumor (T); of those, five had an increase only in tumor size while three presented with an increase in both tumor size and in the number and/or station(s) of lymph nodes (N). Moreover, a total of five patients developed an increase in their stage. More precisely, two patients progressed from T1cN0M0 (IA_3_) to T2bN0M0 (IIA) and one progressed from T2aN0M0 (IB) to T4N0M0 (IIIA), while two of the patients with both tumor and lymph node increase progressed, one from T1aN0M0 (IA_1_) to T1cN1M0 (IIB) and the other one from T1cN0M0 (IA_3_) to T2aN1M0 (IIB). Lymph node progression was from N0 to N1 disease in three patients. Regarding the degree of upstaging, there were two cases that progressed from IA to IIA, two patients progressed from IA to IIB while one patient progressed from IB to resectable IIIA.

However, no significant differences were detected in the increase of the stage of lung cancer between the two groups (Table 2). These differences remained significant also after adjusting for confounders, including age, sex, and lung cancer type (Table 3). Therefore, lung cancer patients during the COVID-19 pandemic waited for treatment for an average period of 41 more days (95% Confidence Interval—C.I. 22 to 60 days), compared to those in the pre-COVID-19 period, after adjusting for confounders. Patients with resectable NSCLC during the COVID-19 era had a 10.7 times higher risk (OR: 10.7 and 95% C.I.: 1.7 to 69.0) of developing an increase in the magnitude of their tumor, compared to the corresponding group before the pandemic. Moreover, there is an indication of a six-times higher risk (OR: 5.9 and 95% C.I. 0.7 to 46.2) of stage increase in lung cancer patients during the COVID-19 pandemic, compared to the pre COVID-19 period, after adjusting for confounders.

## 4. Discussion

This study demonstrated a significant increase in waiting time for surgical treatment in patients with early-stage non-small cell lung cancer (NSCLC) as well as a significant increase in the extent of the disease, as expressed by tumor size (T) and regional lymph node involvement (N) independent of age, gender and lung cancer type. Specifically, our results demonstrate that early-stage lung cancer patients during the COVID-19 pandemic waited for treatment on average 41 more days compared to those in the pre-COVID-19 period. In addition, patients with resectable NSCLC during the COVID-19 era had a 10.7 times higher risk of developing an increase in the size of their tumor, compared to the corresponding group prior to the pandemic. However, no significant change in the stage of lung cancer was found, not unsurprisingly since stage represents a broader group of combinations of diverse tumor size (T) and lymph node involvement (N). However, there was an indication of a six-fold higher risk of stage increase in lung cancer patients during the pandemic, after adjusting for potential confounders. This study indicates that the COVID-19 pandemic had a great impact on the treatment management of NSCLC affecting surgical care.

Diverse clinical guidelines concerning COVID-19 management in cancer patients have been recently published providing some guidance for the standard anticancer treatments of lung cancer patients. More particularly, the Centers for Disease Control and Prevention (CDC) has suggested that all elective surgeries should be rescheduled, while the American Society of Clinical Oncology recommended that cancer healthcare professionals should decide on patient management during the pandemic on an individual-by-individual basis, taking into consideration the potential risks due to delayed cancer-related surgical resections [17]. In addition, the American College of Surgeons proposed a case triage-based guideline regarding surgical care according to available hospital resources and phases of the COVID-19 pandemic [17]. However, thus far, no conclusive and universally accepted recommendations exist regarding the application of surgical treatment in resectable NSCLC during the pandemic [17]. Similarly, the exact impact that the pandemic had on the delivery of surgical care in this group of patients remains unclear [17]. In addition, limited and contradictory data have been published about the incidence of post-operative SARS-CoV-2 infection and mortality in patients who underwent resection for lung cancer [18,19]. To the best of our knowledge, this is the first study investigating the impact of COVID-19 on resectable lung cancer during the pandemic, providing an indication of the serious consequences in the surgical therapeutic care.

Our results regarding the effect that the COVID-19 pandemic had on the surgical care are compatible with previous studies in the oncology field which also reveal a significant impact on the associated medical care in patients with cancer [20,21]. In a nationwide survey among patients with cancer conducted in the Netherlands, it was shown that 30% of cancer patients reported consequences in oncological treatment and follow-up [20]. Moreover, it was demonstrated that chemotherapy and immunotherapy were the most frequently deferred (or postponed) therapies [20]. This is in accordance with our results, demonstrating a significant increase in waiting times for surgical treatment in patients with early-stage NSCLC. Concomitantly with delays and discontinuation of treatment, various studies have also revealed a hesitancy by cancer patients to consult and visit hospitals for non-COVID-19 issues during the pandemic [20,21]. The observed reduction in the number of consultations for cancer symptoms in primary care during the pandemic may further lead to more advanced cases of lung cancer that unfortunately will not be eligible for surgical treatment [22,23]. Finally, another critical consequence is the temporary discontinuation of screening programs for cancer that has contributed to a significant decrease in the incidence of new cancer cases [21,24]. All the aforementioned data confirm the need for lung cancer multidisciplinary teams to balance the risk of patients to SARS-CoV-2 infection with the need for optimal effective treatment.

Concerning recommendations on surgical treatment of early-stage lung cancer during the pandemic, data are contradictory It has been generally proposed that elective surgeries should be rescheduled [25,26,27]; however, contrarily, the European Association of Medical Oncology recommends keeping all surgeries as a priority in the management of early NSCLC and that surgical delays should not exceed six to eight weeks [28]. It is widely accepted that patients undergoing surgical treatment within 12 weeks of diagnosis have considerably improved overall survival than those with delayed treatment delivery of more than 12 weeks [15]. The significant increase in waiting time for surgical treatment observed in our study and the subsequent increase of the extent of the disease could represent crucial elements, that must of necessity be taken into consideration for potential future pandemics. In this direction, the results of our study underline the importance of on-time surgical treatment delivery during the pandemic, especially on early-stage NSCLC. It is well established that complete surgical resection in early-stage NSCLC remains the primary therapeutic approach, presenting higher survival rates than other treatment modalities [29]. Finally, previous studies have demonstrated that for each week of surgical delay beyond 12 weeks, the risk for recurrence and reduced survival increases [15,30,31,32]. So far, data regarding the effect of COVID-19 and of our decisions on early-stage NSCLC patients eligible for surgical treatment are lacking. These results reveal the pandemic’s significant impact on surgical care leading to considerable delays in treatment and a consequent increase in tumor size, providing evidence for the need to continue delivering prompt surgical treatment in early stage NSCLC even during the COVID-19 pandemic. This pandemic may soon be controlled; however, future similar outbreaks are likely to occur.

This study has a number of limitations, the most important being the moderate number of patients analyzed. In addition, due to the design, aims, and short study period, it was not possible to assess the impact of the pandemic on disease-free survival and overall mortality of early-stage NSCLC patients. Nevertheless, although our study represents a single-academic center study, it is based on a well-defined group of early-stage NSCLC patients and is one of the few studies thus far that have investigated the impact of COVID-19 pandemic on resectable lung cancer. Considering also the large number of patients dying from lung cancer during the pandemic [3], the importance of our results support that the need to continue timely delivery of surgical treatment in early-stage NSCLC is of critical significance. Therefore, the clinical message from our results is to highlight the need for prompt surgical treatment in early-stage NSCLC, keeping all surgeries as a priority, thus optimizing cancer care delivery in future pandemics.

## 5. Conclusions

The COVID-19 pandemic has had a significant impact on surgical care, leading to significant delays in treatment and an increase in tumor size in resectable NSCLC patients independent of age, gender and lung cancer type. Considering the large number of patients dying from lung cancer during the pandemic, continuing with the prompt delivery of surgical treatment in early-stage NSCLC is critical. Appropriate changes in the treatment decisions and management of lung cancer patients should be proposed during this COVID-19 pandemic, and also in future pandemics.

## Figures and Tables

**Table 1:** Demographic and clinical characteristics of lung cancer patients before and during the pandemic COVID-19.

	Patients with Resectable NSCLC		
Demographic Characteristics	Total (n = 61)	pre COVID-19 (Control Group) (n = 33)	COVID-19 (Study Group) (n = 28)
Age (mean, SD: years)	67.0 (7.3)	67.1 (7.5)	67.0 (7.1)
Sex (n, %: males)	50.0 (82.0)	28.0 (84.8)	22.0 (78.6)
Clinical Characteristics			
Days waiting treatment (median, IQR)	23.0 (14.0–37.0)	18.0 (11.0–23.0)	47.0 (23.0–100.0)
Increase of the size (n, %: yes)	10.0 (16.4)	2.0 (6.1)	8.0 (28.6)
Increase of the stage (n, %: yes)	7.0 (11.5)	2.0 (6.1)	5.0 (17.9)
Death (n, %: yes)	1.0 (1.6)	0.0 (0.0)	1.0 (3.6)
Type of cancer (n, %)			
Squamous	28.0 (45.9)	13.0 (39.4)	15.0 (53.6)
Adenocarcinoma	29.0 (47.5)	17.0 (51.5)	12.0 (42.9)
Other	4.0 (6.6)	3.0 (9.1)	1.0 (3.6)

**Table 2:** Comparison of the characteristics of lung cancer patients before and during the COVID-19 pandemic.

Lung Cancer Patients	Aσθενείς		
Clinical Characteristics	pre COVID-19 (n = 33)	COVID-19 (n = 28)	*p*-Value
Days waiting treatment (median, IQR)	18 (11–23)	47 (23–100)	<0.001 ^1^ *
Increase of the size (n, %: yes)	2 (8.7)	8 (44.4)	0.012 ^2^ *
Increase of the stage (n, %: yes)	2 (8.7)	5 (27.8)	0.209 ^2^

^1^ Mann-Whitney U test. ^2^ Fisher’s exact test. * Statistically significant result at α = 5%.

**Table 3:** Results from multiple linear regression model (b-coefficient & 95% Confidence Interval) and multiple logistic models (Odds Ratio & 95% Confidence Interval) assessing the difference between the 2 groups of lung cancer patients (during COVID-19 pandemic vs. before the COVID-19 pandemic), with dependent variable in: Model 1) waiting time for surgical treatment, Model 2) possibility of increase of the size of tumour and Model 3) possibility of increase of the stage of lung cancer, after further adjusting for age, sex, lung cancer type.

	Patient Group	*b-coef*	95% C.I. for *b-coef*	*p*-Value
Model 1	Pre COVID-19	Reference category		
	COVID-19	40.8	(21.9 to 59.6)	<0.001 ^1^ *
		OR	95% C.I. for OR	*p*-Value
Model 2	Pre COVID-19	Reference category		
	COVID-19	10.7	(1.7 to 69.0)	0.012 ^2^ *
Model 3	Pre COVID-19	Reference category		
	COVID-19	5.9	(0.7 to 46.2)	0.093 ^2^

^1^ Dependent variable: Time waiting for treatment (days). ^2^ Dependent variable: Increase of the tumor size (yes vs. no). ^3^ Dependent variable: Increase of stage (yes vs. no). b-coef: beta coefficient for patient group (COVID-19 vs. pre COVID-19). OR: Odds Ratio for patient group COVID-19 vs. pre COVID-19). 95% C.I.: 95% Confidence Interval. * Statistically significant result at α = 5%.

## Data Availability

The data presented in this study are available in this article.

## Abbreviations

Coronavirus disease (COVID-19), Severe acute respiratory syndrome coronavirus 2 (SARS-CoV-2), Non-small cell lung cancer (NSCLC), standard deviation (SD), Median and interquartile range (IQR)
